# Efficacy of Administration of an Angiotensin Converting Enzyme Inhibitor for Two Years on Autonomic and Peripheral Neuropathy in Patients with Diabetes Mellitus

**DOI:** 10.1155/2017/6719239

**Published:** 2017-03-08

**Authors:** Triantafyllos Didangelos, Konstantinos Tziomalos, Charalambos Margaritidis, Zisis Kontoninas, Ioannis Stergiou, Stefanos Tsotoulidis, Eleni Karlafti, Alexandros Mourouglakis, Apostolos I. Hatzitolios

**Affiliations:** Diabetes Center, First Propedeutic Department of Internal Medicine, Medical School, Aristotle University of Thessaloniki, AHEPA Hospital, Thessaloniki, Greece

## Abstract

*Aim*. To evaluate the effect of quinapril on diabetic cardiovascular autonomic neuropathy (CAN) and peripheral neuropathy (DPN).* Patients and Methods*. Sixty-three consecutive patients with diabetes mellitus [43% males, 27 with type 1 DM, mean age 52 years (range 22–65)], definite DCAN [abnormal results in 2 cardiovascular autonomic reflex tests (CARTs)], and DPN were randomized to quinapril 20 mg/day (group A, *n* = 31) or placebo (group B, *n* = 32) for 2 years. Patients with hypertension or coronary heart disease were excluded. To detect DPN and DCAN, the Michigan Neuropathy Screening Instrument Questionnaire and Examination (MNSIQ and MNSIE), measurement of vibration perception threshold with biothesiometer (BIO), and CARTs [R-R variation during deep breathing [assessed by expiration/inspiration ratio (E/I), mean circular resultant (MCR), and standard deviation (SD)], Valsalva maneuver (Vals), 30 : 15 ratio, and orthostatic hypotension (OH)] were used.* Results*. In group A, E/I, MCR, and SD increased (*p* for all comparisons < 0.05). Other indices (Vals, 30 : 15, OH, MNSIQ, MNSIE, and BIO) did not change. In group B, all CART indices deteriorated, except Vals, which did not change. MNSIQ, MNSIE, and BIO did not change.* Conclusions.* Treatment with quinapril improves DCAN (mainly parasympathetic dysfunction). Improved autonomic balance may improve the long-term outcome of diabetic patients.

## 1. Introduction

Diabetes mellitus (DM) is the most common cause of neuropathy and diabetic neuropathy (DN) comprises a heterogeneous group of disorders that can cause neuronal dysfunction throughout the human body. The Toronto Consensus Panel on DN divided in 2010 the disease into typical and atypical neuropathy [1] Typical diabetic peripheral neuropathy (DPN) is “a symmetrical, length-dependent sensorimotor polyneuropathy attributable to metabolic and microvascular alterations as a result of chronic hyperglycemia exposure and cardiovascular risk covariates.” Atypical forms of DN differ in onset, course, manifestations, associations, and putative mechanisms and are likely to be associated with pain and/or dysautonomia. Peripheral and autonomic neuropathies are the most common manifestations of DN, which often coexist. Diabetic peripheral neuropathy is the second most common form of DN and is estimated to affect 45–50% of all patients with DM [2]. The prevalence varies according to the severity and duration of hyperglycaemia but overall polyneuropathy is present in up to 50% of people with long-standing DM [3].

DPN represents a major health problem as it may present with excruciating neuropathic pain and is responsible for substantial morbidity, resulting from foot ulceration, amputations, and impaired quality of life, as well as with increased mortality. The manifestations of diabetic autonomic neuropathy (DAN) are manifold affecting all systems and organs innervated from autonomic system, but cardiovascular, urogenital, gastrointestinal, pupillomotor, thermoregulatory, and sudomotor systems are the most important. Diabetic cardiovascular autonomic neuropathy (DCAN) is characterized by autonomic dysfunction of the cardiovascular system. It is the most prevalent and well-studied form of DAN [4]. It is characterized by alterations in the control of heart rate and vascular hemodynamics. The prevalence of DCAN ranges from 2.5 to 50% in different cohorts. The prevalence of confirmed DCAN is around 20% and rises up to 65% with age and DM duration. DCAN has been shown to negatively impact mortality due to its relationship with serious comorbidities (including silent myocardial ischemia, coronary heart disease (CHD), stroke, diabetic nephropathy, and increased perioperative morbidity) [4]. Thus, the management of DCAN has important implications for the prognosis of DM.

Despite the significant individual and social burden associated with diabetic neuropathy, its treatment remains unsatisfactory. This is in part due to the innately unpredictable and complex nature of the disease, combined with limited systematic diagnostic testing, which differs from diabetic retinopathy and nephropathy, where the disease is more predictable and the diagnostic tests more straightforward. In the current study, we chose to use the most valid and accurate diagnostic tests [Michigan Neuropathy Screening Instrument Questionnaire and Examination (MNSIQ and MNSIE) and Cardiovascular Reflex Tests (CRTs)] for the evaluation of neuropathy in well characterized and highly selected patients. Moreover, there are currently no FDA-approved therapies for diabetic neuropathy and only 3 approved therapies for painful DPN. No treatment results in complete resolution of the underlying pathophysiological abnormalities and treatment of DN is an unmet need in clinical practice. Only strict metabolic control appears to have a beneficial effect on the prevention and delay of the onset of DN and to reduce the prevalence of established DN [5–8].

The aim of the present study was to evaluate the efficacy and safety of the administration of an angiotensin converting enzyme (ACE) inhibitor, quinapril 20 mg/day, for two years on DCAN and DPN in patients with type 1 and 2 DM.

## 2. Research Design and Methods

### 2.1. Patient Selection

This open, parallel-group, controlled study included 63 adult patients with long-standing types 1 and 2 DM, who were recruited from the outpatient diabetes clinics in AHEPA University hospital and Hippokration hospital of Thessaloniki, Greece. The study was approved by the institutional ethics committee and all subjects gave written informed consent. The study started in 1999. All patients were asymptomatic, had a normal electrocardiogram, and were normotensive (blood pressure ≤ 130/85 mmHg). They also had normal renal function and were under no medication other than insulin. CHD was excluded on the basis of normal thallium 201 myocardial perfusion imaging. All patients were well characterized and highly selected. Patients were randomized to receive either quinapril 20 mg/day (group A, *n* = 31) or no treatment (group B, *n* = 32). Demographic characteristics of the patients are shown in Table 1.

### 2.2. Cardiovascular Autonomic Reflex Tests (CARTs)

The Monitor ONE NDX device (QMED Industries, Clark, NJ, USA) was used for the measurement of the autonomic nervous function (ANF) indices. ANF was assessed according to the consensus statement of the American Diabetes Association and the American Academy of Neurology [9] and the Toronto Consensus Panel on Diabetic Neuropathy [10] taking into account various factors such as concomitant illnesses and lifestyle (exercise, hypoglycemia, smoking, and caffeine intake). The following tests were performed as previously described: (1) beat to beat variation of R-R interval assessed by (a) expiration/inspiration index (E/I Index), (b) mean circular resultant (MCR) vector analysis (probably the most reliable ANF index), and (c) standard deviation (SD) and (2) Valsalva maneuver (Valsalva Index), (3) variation of R-R interval during postural change (30 : 15 Index), and (4) variation of systolic blood pressure during postural change (standing). The presence of definite DCAN was established if at least 2 of the above-mentioned CARTs were abnormal. The normal values adopted were those reported by Ziegler et al. [11].

### 2.3. Michigan Neuropathy Screening Instrument (MNSI)

MNSI has 2 steps to assess history of neuropathic symptoms and physical examination to evaluate the appearance and sensation of feet. An objective test with 4 questions included foot skin inspection for deformities, dry skin, calluses, infections, fissures and ulcer, ankle reflex, and vibration sensation tested by a 128 HZ tuning fork placed over great toe (MNS1Q). The test was performed by an experienced physician. A score ≥ 2 was considered abnormal. Abnormality in each item is graded between 0.5 and 1 and at least more than 2 abnormal items are needed to reach the score of neuropathy [12].

All tests were performed in the same day by an experienced physician blinded to the treatment. All patients had both definite DCAN (2 or more CARTs abnormal) and definite DPN.

## 3. Statistical Analysis

All data were analyzed with the statistical package SPSS (version 17.0; SPSS, Chicago, IL, USA). Data are presented as percentages for categorical variables and as mean and standard deviation for continuous variables. Differences in categorical variables between groups at baseline were assessed with the chi-square test. Differences in continuous variables between groups at baseline and at the end of follow-up were assessed with the independent samples *t*-test. Paired samples *t*-test was used for comparisons of DPN and DCAN indices between and after treatment. In all cases, a two-tailed *p* < 0.05 was considered significant.

## 4. Results 

After 2 years of follow-up, improvement was recorded in group A in all indices of deep breathing test (E/I, MCR, SD) versus baseline (Table 2). The other indices, Valsalva Index, 30 : 15, and orthostatic hypotension did not change versus baseline (Table 2).

In group B, all indices displayed significant deterioration in comparison to baseline at month 24 of follow-up except the Valsalva Index that remained unchanged throughout the study (Table 2).

At the end of follow-up, all indices in group A, except Valsalva Index, were better than in group B (Table 2).

All indices of DPN did not change during the study in either group A or group B and did not differ between the 2 groups at the end of follow-up (Table 3).

## 5. Discussion

The present study demonstrated for the first time that treatment with quinapril for 2 years improves parasympathetic function of DCAN as expressed with the indices of deep breathing test. We did not observe any significant improvement or deterioration in DPN, according to MNSI and BIO indices.

In the present study, we used CARTs, MNSI, and BIO as the most valid and appropriate tests to diagnose definite neuropathy. This study design was adopted because we wanted to assess the clear effect of ACE inhibition on DCAN and DPN without interference of any other disease except DM or any potential drug-induced change in autonomic nervous system function parameters. Despite the fact that many new methods have been described for the diagnosis of DCAN such as heart rate variability, metaiodobenzylguanidine scan, and corneal confocal microscopy, they are not included in the criteria for the diagnosis of DCAN according to the San Antonio conference and the new proposal from the Toronto panel. The criteria suggested from these 2 conferences are that 2 or more of the following cardiovascular reflex tests should be abnormal: (1) deep breathing test, (2) Valsalva maneuver, (3) 30 : 15 index, and (4) orthostatic hypotension.

The present study included only well characterized, highly selected normotensive patients with definite diagnosed DCAN and DPN free of CHD (based on a normal scintigraphy test), diabetic cardiomyopathy, nephropathy, arrhythmias, or heart failure of any etiology. In our previous study with a 6-month duration, we found an improvement in indices of 24 h HRV without any change in indices of CARTs [13]. In another study from our group, we also observed after 1 year of treatment with quinapril improvement of DCAN and left ventricular dysfunction [14]. However, the former study was criticized because of lack of control group. The Steno-2 trial reported that a multifactorial cardiovascular risk intervention (including ACE inhibition) appeared to reduce the prevalence of autonomic dysfunction by 63% [15]. In the former study, glucose-lowering therapy appeared to have the least impact in preventing DCAN compared with antihypertensive drugs, lipid-lowering agents, antiplatelet therapy, and vitamin and mineral supplementation [15]. In another recent study, patients received a-lipoic acid plus ACE inhibition and there was an improvement in DCAN after 4 years of treatment [16]. Therefore, it is difficult to evaluate the effect of ACE inhibition in the latter two studies, since other treatments were also administered. To the best of our knowledge, the current study is the first that shows the effects of monotherapy with an ACE inhibitor on DCAN.

At baseline, the values of most standard CARTs were below the lower limit of normal. The values of parasympathetic related tests were more adversely affected at baseline than those of sympathetic related tests. It is argued that indices of the deep breathing test (E : I index, SD, and MCR of R-R intervals), considered to be related to vagal tone, were negatively affected and are the earliest markers of DCAN deterioration. So, maybe, they are the first to improve with appropriate treatment, as observed in the current study. Valsalva maneuver is a more complex test; it encompasses a complex reflex arc involving both sympathetic and vagal pathways to the heart, sympathetic pathways to the vascular tree, and baroreceptors in the chest and lungs [11]. Thus, it is reasonable that the Valsalva Index is affected after total and significant autonomic nervous system entanglement, which probably occurs later than the 2 year follow-up period of our study.

Indices of DPN did not change during the 2-year follow-up period. Only 2 randomized, double-blind, placebo-controlled studies evaluated this topic. One trial from Malik et al. reported that peroneal nerve motor conduction velocity increased after 12 months of treatment with trandolapril compared with placebo [17]. Vibration perception threshold, autonomic function and the neuropathy symptom, and deficit score showed no improvement in either group. Our experience with administration of quinapril for 6 months in a randomized, double-blind, placebo-controlled trial was an improvement in indices of 24 h HRV with no change in vibration perception threshold. But, in the study by Malik et al., values from indices of CARTs were much lower than in ours and in our study indices of CARTs during 6 months of treatment did not improve and we did not evaluate nerve conduction velocity. The obvious question is why no pathogenetic treatment for DPN has proved sufficiently efficacious to achieve regulatory approval. Ziegler and Luff suggested that trials were hampered by a generally poor design and short follow-up and by being limited to patients with advanced DPN [18]. They suggested that trials involving patients with early DPN, conducted over 3–5 years to establish a delay or arrest in the progression of neuropathy, rather than reversal, were more likely to be successful [18].

Previous studies in experimental models [19, 20] have shown a reduction in the progression of DN. These effects are mainly mediated through the vasodilating properties of ACE inhibitors, when used for improvement of nerve flow velocity. A beta blockade-like effect of quinapril was observed in a previous study; that is, quinapril compared with lisinopril decreased the heart rate (−12%, *p* < 0.01) in patients with mild to moderate hypertension [21]. In the lisinopril group, no change in heart rate was observed [21]. So, quinapril appears to combine beta blockade-like and ACE inhibition properties, without the side effects of b-blocking agents. This was the reason why we chose to use in the current study quinapril among other ACE inhibitors.

Moreover, formation of advanced glycosylated end products (AGEs) in DM appears to play a crucial role in the pathogenesis of microvascular complications and maybe in the “metabolic memory” observed in large studies. It has been proposed that the pathophysiological cascades triggered by AGEs have a dominant, hyperglycemia-independent role in the onset of the microvascular complications of diabetes [22]. Furthermore, ACE inhibition, in experimental trials, reduces the accumulation of AGEs in DM [23, 24] and maybe that is another mechanism of action of these drugs against the development of microvascular complications in DM. Moreover, a beneficial effect of ACE inhibitors has been suggested in many studies on retinopathy and nephropathy.

Data supporting the role of glycemic control in both the primary and secondary prevention of DPN in patients with type 1 DM comes from the Diabetic Control and Complications Trial (DCCT) [5]. In the intensive glucose control arm, a 60% reduction in the incidence of DPN and a 45% reduction in the incidence of DCAN were observed [5]. In the Epidemiology of Diabetes Interventions and Complications (EDIC) study, despite no difference in glycemic control, the prevalence and incidence of DPN and CAN were significantly reduced in patients who received prior intensive insulin treatment compared with patients who received standard insulin therapy during the DCCT [5]. This protective effect of prior intensive glycemic control, termed metabolic memory, persisted until 13 to 14 years after the end of the DCCT [5]. For CAN, differences in glycated hemoglobin levels during the DCCT explained almost all the protective effects of intensive versus standard therapy on the risk of incident CAN, supporting early commencement of intensive treatment in T1D [5]. In T2DM, there is less evidence of benefits of intensive glycemic control on DN. The United Kingdom Prospective Diabetes Study (UKPDS) emphasized the impact of glycemic control on microvascular complications in type 2 DM and reported a lower rate of impaired vibration perception threshold (VPT) with intensive therapy versus standard therapy, even though this effect became significant only after 15 years of follow-up (relative risk 0.60, 95% confidence interval 0.39–0.94) [25]. Furthermore, a Cochrane review of 17 randomized trials concluded that strict glycemic control prevented neuropathy in patients with type 1 DM but a trend towards reduced incidence in type 2 DM was not significant [26].

Our group proposed for the first time the management of DCAN with ACE inhibitors and reported an increase in indices of 24 h HRV, which have been considered as the earliest markers of autonomic dysfunction [13]. In the present study, we report a clear effect of quinapril in well characterized patients with types 1 and 2 DM with definite DCAN and DPN.

Another drug class that has been studied in the context of DCAN and DPN is aldose reductase inhibitors (ARIs). In a previous study from our group, tolrestat, an ARI, improved indices of CARTs in patients with definite DCAN after 2 years of treatment [27]. In a meta-analysis, Hu et al. evaluated the efficacy and safety of ARIs for the treatment of CAN in DM, based on CARTs [28]. From their analysis of 10 studies, the authors concluded that ARIs improved cardiac autonomic function [28].

Additional data for the beneficial effect of ACE inhibition on DCAN were reported in the NATHAN-1 trial [16]. The authors used as efficacy measures the Neuropathy Impairment Score of the lower limbs (NIS-LL) and heart rate during deep breathing (HRDB) [16]. Participants treated with a-lipoic acid for 4 years who received ACE inhibitors showed a greater improvement in HRDB after 4 years [16].

Many other drugs have been used in the management of DN and further studies are necessary for identifying the best combinations of treatments for diabetic neuropathy [29].

Foot problems from underlying DN are a major cause for developing ulcers, Charcot foot abnormalities, injuries, infections, and lower extremity amputation and this is a lifetime risk for patients with DM. As neuropathy progresses, impairment of body balance and gait abnormalities may be encountered and all these in addition to motor dysfunction may predispose to falls and fractures. Moreover, DCAN could predispose to these adverse events. The economic cost from foot problems is big worldwide and in Greece [30]. Patients with diabetic neuropathy should be routinely counseled about their disease, in particular focusing on patient concerns and expectations. Moreover, the role of strict glycemic control should be emphasized. Thus, patients should be advised on the need for meticulous foot hygiene, appropriate footwear, and mobility support as needed.

In conclusion, in the present study, DCAN (mainly parasympathetic dysfunction) improved after 2 years of treatment with quinapril. Improved autonomic balance may be of clinical importance in the long-term outcome of patients with DM. A clear effect of quinapril on DCAN has been demonstrated. Strict glycemic control is the only confirmed treatment for prevention and delaying the development of diabetic neuropathy today. ACE inhibition and especially quinapril could be an alternative tool for the treatment of DN and the beneficial effect could be more prominent if the treatment begins at the early stages of neuropathy.

## Figures and Tables

**Table 1:** Characteristics of patients at baseline.

	Group Α (*n* = 31)	Group Β (*n* = 32)	*p*
Age (years)	52.7 ± 16.4	51.9 ± 13.9	NS
Males (%)	48.4	37.5	NS
Diabetes mellitus duration (years)	17.8 ± 7.4	18.1 ± 8.2	NS
Type 1 diabetes mellitus (%)	45.2	40.6	NS
HbA_1c_ (%)	7.1 ± 2.2	7.2 ± 2.4	NS
Fasting plasma glucose (mg/dl)	125 ± 27	129 ± 19	NS
Total cholesterol (mg/dl)	189 ± 37	193 ± 51	NS
High density lipoprotein cholesterol (mg/dl)	46 ± 10	45 ± 10	NS
Low density lipoprotein cholesterol (mg/dl)	107 ± 36	118 ± 49	NS
Triglycerides (mg/dl)	185 ± 70	171 ± 68	NS
Creatinine (mg/dl)	0.97 ± 0.19	0.98 ± 0.25	NS
Estimated glomerular filtration rate (ml/min/1.73 m^2^)	112 ± 34	109 ± 37	NS
Uric acid (mg/dl)	5.4 ± 1.3	6.1 ± 2.1	NS

**Table 2:** Changes in cardiovascular autonomic reflex tests during the study in patients who received quinapril (group A) and in those who did not (group B).

	Group A (*n* = 31)			Group B (*n* = 32)			*p* (group A versus group B at end of treatment)
	Baseline	End of treatment	*p* (versus baseline)	Baseline	End of treatment	*p* (versus baseline)	
E/I index	1.11 ± 0.06	1.23 ± 0.12	0.011	1.09 ± 0.06	1.04 ± 0.04	0.007	<0.001
MCR	18.1 ± 6.2	38.7 ± 20.5	0.006	14.2 ± 4.2	8.1 ± 4.5	0.01	<0.001
SD	31.1 ± 11.9	56.6 ± 23.0	0.004	28.2 ± 9.9	15.5 ± 7.4	<0.05	<0.001
Valsalva index	1.48 ± 0.28	1.56 ± 0.33	NS	1.52 ± 0.22	1.50 ± 0.28	NS	NS
30 : 15 index	1.15 ± 0.12	1.18 ± 0.12	NS	1.15 ± 0.06	1.08 ± 0.04	<0.05	<0.001
OH	16.0 ± 11.8	10.4 ± 6.1	NS	18.5 ± 4.5	28.0 ± 6.3	0.018	<0.001

E/I: expiration/inspiration; MCR: mean circular resultant; SD: standard deviation; OH: orthostatic hypotension.

**Table 3:** Changes in indices of diabetic peripheral neuropathy during the study in patients who received quinapril (group A) and in those who did not (group B).

	Group A (*n* = 31)			Group B (*n* = 32)		
	Baseline	End of treatment	*p* (versus baseline)	Baseline	End of treatment	*p* (versus baseline)
Michigan Neuropathy Screening Instrument	2.6 ± 0.4	2.4 ± 0.3	NS	2.4 ± 0.4	2.5 ± 0.5	NS
Vibration perception threshold	23 ± 8	20 ± 7	NS	25 ± 8	24 ± 9	NS

## Additional Points

*Novelty Statement.* (i) The present study shows the efficacy and safety of treatment with angiotensin converting enzyme (ACE) inhibitors in patients with cardiovascular autonomic neuropathy (CAN), especially in patients with type 2 diabetes mellitus at early stages of the disease, in whom coronary heart disease and hypertension are much more common than in type 1 diabetes mellitus. (ii) Moreover, many studies suffer from inadequate definition of diabetic cardiovascular autonomic neuropathy (DCAN) or supporting data for the presence of DCAN. (iii) The present study explores in highly selected patients this association and is the first to report an improvement in DCAN after treatment with an ACE inhibitor for 2 years.
